# Supplementary material for: The causal relationship between the human gut microbiota and pyogenic arthritis: a Mendelian randomization study
Source: Front Cell Infect Microbiol. 2024 Nov 26;14:1452480. doi: 10.3389/fcimb.2024.1452480 (PMC11629706; doi:10.3389/fcimb.2024.1452480)
Supplement: Supplementary file 1 [file DataSheet1.zip › mendelian test/ebi-a-GCST90027569.csv_leave_one_out.pdf]

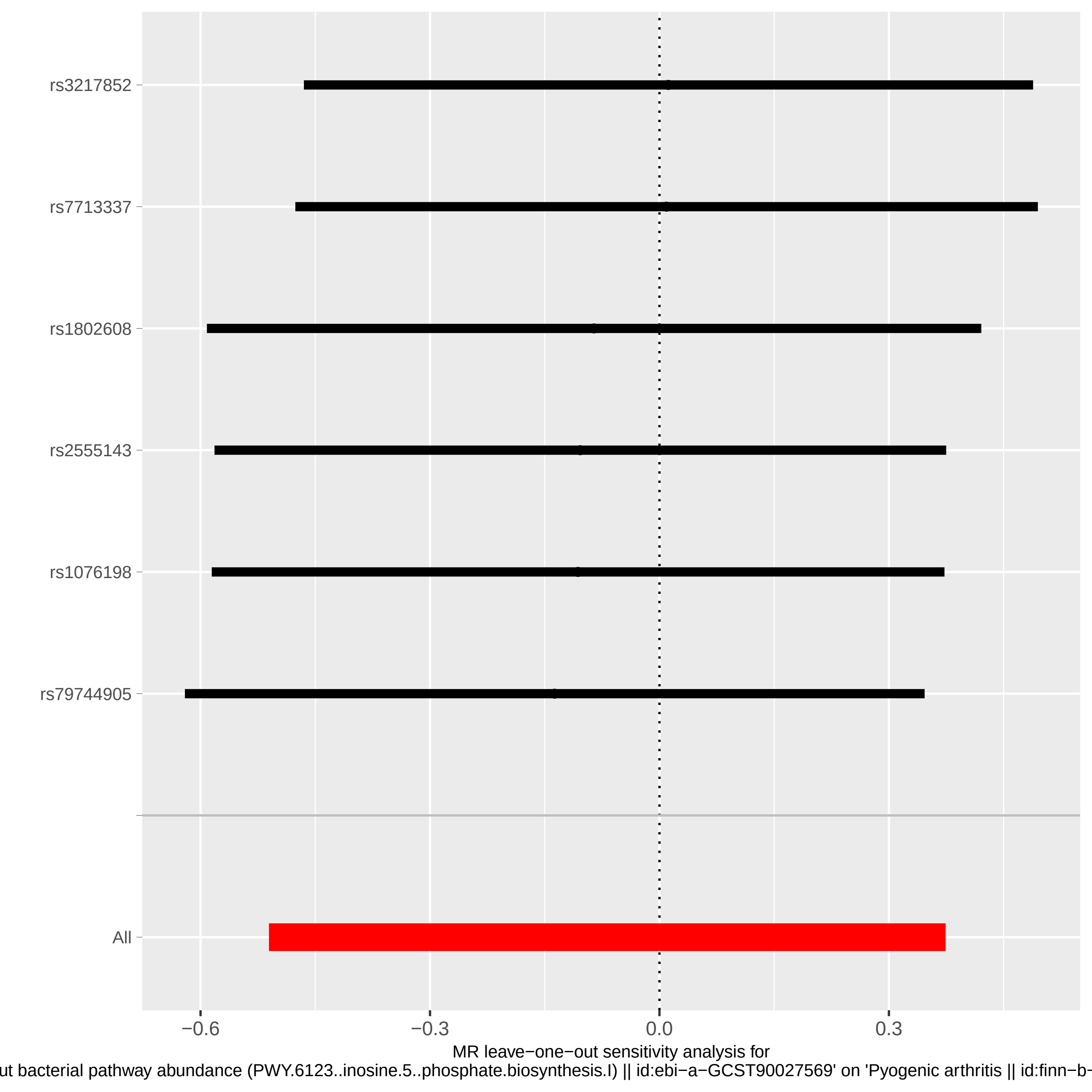
rs3217852
rs7713337
rs1802608
rs2555143
rs1076198
rs79744905
All
−0.6
−0.3
0.0
0.3
MR leave−one−out sensitivity analysis for
ut bacterial pathway abundance (PWY.6123..inosine.5..phosphate.biosynthesis.I) || id:ebi−a−GCST90027569' on 'Pyogenic arthritis || id:finn−b−
